# Tuning Molecular Orientation Responses of Microfluidic Liquid Crystal Dispersions to Colloid and Polymer Flows

**DOI:** 10.3390/ijms241713555

**Published:** 2023-08-31

**Authors:** Artem Bezrukov, Yury Galyametdinov

**Affiliations:** Department of Physical and Colloid Chemistry, Kazan National Research Technological University, 68 Karl Marx Str., Kazan 420015, Russia; yugal2002@mail.ru

**Keywords:** liquid crystals, microfluidics, dispersion, molecular orientation, molecular diagnostics, ordering, colloids, polymer solutions, surfactants

## Abstract

An important approach to molecular diagnostics is integrating organized substances that provide complex molecular level responses to introduced chemical and biological agents with conditions that optimize and distinguish such responses. In this respect, liquid crystal dispersions are attractive components of molecular diagnostic tools. This paper analyzes a colloid system, containing a nematic liquid crystal as a dispersed phase, and aqueous surfactant and polymer solutions as the continuous phases. We applied a microfluidic approach for tuning orientation of liquid crystal molecules in picoliter droplets immobilized on microchannel walls. Introduction of surfactant to the aqueous phase was found to proportionally increase the order parameter of liquid crystal molecules in microdroplets. Infusion of polymer solutions into surfactant-mediated microfluidic liquid crystal dispersions increased the order parameter at much lower surfactant concentrations, while further infusion of surfactant solutions randomized the orientation of liquid crystal molecules. These effects were correlated with the adsorption of surfactant molecules on surfaces of microdroplets, stabilizing the effect of a polymer matrix on bound surfactant ions and the formation of insoluble polymer–colloid aggregates, respectively. The revealed molecular behavior of liquid crystal dispersions may contribute to optimized synthesis of responsive liquid crystal dispersions for in-flow molecular diagnostics of polymers and colloids, and the development of functional laboratory-on-chip prototypes.

## 1. Introduction

Organized media and nanoscale systems play an increasingly significant role in molecular sensing due to their abilities to perform specific interactions with various chemical agents [1,2,3,4]. A practically important research focus in this area is to develop systems which generate an easily detectable response to occurring molecular-level processes, such as changes in optical properties or luminescence [5,6,7,8].

Liquid crystals (LCs) are optically active anisotropic media, which are sensitive to a variety of substances due to an intrinsic ordering mobility of LC molecules in the presence of a directing factor [9,10,11]. Interactions between LC molecules in droplets or films and solutes in the contacting aqueous phase were reported to result in changes in LC optical properties in the presence of glucose [12], inorganic ions [13] and organic molecules [14,15,16], varying temperature and pH conditions [17,18], as well as amphiphiles [1,19,20] and polymers [9,21,22].

Microfluidics offers a precise dynamic control of both a liquid crystal phase [23,24,25] and an aqueous media with dissolved substances [26,27,28,29]. Laminar flows of media in microchannel confinement represent an ordered non-equilibrium environment [30], suitable for multiple operations of chemical reactions, analysis and imaging in so-called lab-on-chip devices [31,32] and organ-on-chip prototypes [33]. Microfluidic devices are garnering attention for fabrication of functional LC-based systems, which can be used for sensing applications [6,34,35,36] and synthesis of drug delivery systems from polymers and colloids [37,38,39,40]. Therefore, microfluidic chips with an integrated LC matrix and contacting aqueous solutions of polymers and amphiphiles represent a fruitful research area in colloid and interface science and microfluidic technology, with an application potential in sensing various molecular agents of chemical and biochemical nature.

A related trend in developing microfluidic devices with an integrated LC matrix is generating liquid crystal dispersions in microchannels [1,6,12,19,41]. LC microdroplets are easy to produce with conventional flow focusing microchips [42,43], without modifications of microchannel surfaces required for creating microfluidic LC films on microchannel surfaces [5,34,44]. Optical properties of LC microdroplets are particularly sensitive to surfactants in the contacting aqueous phase, which adsorb on the LC surface and align the LC molecules perpendicular to the LC-water interface [11,17,19,45]. On the other hand, surfactants are capable of actively interacting with polymers, which make them convenient and popular intermediates for anchoring polymer macromolecules to LC microdroplets and detecting the resulting changes in the ordering of LC molecules [9,46,47].

A fruitful approach to developing microfluidic sensing systems is to process microchannel surfaces with various substances [48,49]. This approach is relevant to microfluidic LC films [9,34]. Microfluidic LC droplets are, however, mostly studied in non-immobilized conditions [12,15,17,18,19,41,47] and publications on immobile LC droplets on microchannel surfaces are relatively limited [46,50]. Orientation transitions of LC molecules are mostly discussed qualitatively, while similar papers on deposited LC films provide a quantitative analysis of specific molecular orientation aspects such as tilt angles of LC molecules [20,21,51]. In this respect, immobilized LC microdroplets can be potentially considered as LC phase fragments immobilized on a microchannel surface. They may be suitable for a similar quantitative analysis of LC molecular orientation responses to variable microflows of polymers and amphiphiles.

This paper aims to study the generation of LC microdroplets immobilized on microchannel walls and characterize the ordering of LC molecules in the presence of polymer macromolecules and amphiphiles introduced in aqueous microflows. We study the impact of surfactant concentration on the orientation of LC molecules in droplets and calculate their tilt angles and ordering parameters. We introduce polymer solutions to surfactant-mediated LC droplets in different concentrations, and sequence and characterize respective changes in the ordering of LC molecules. Finally, we analyze the behavior of immobilized LC droplets during transverse diffusion of polymer macromolecules and compare it with predictions of the microfluidic diffusion model.

## 2. Results

### 2.1. Comparative Characterization of the LC-Polymer Surfactant System

**Figure 1:**
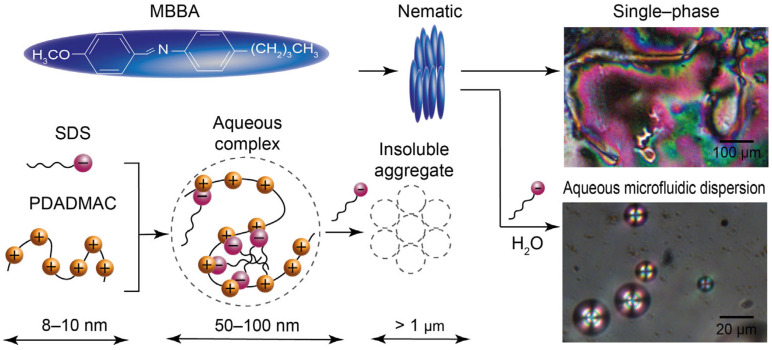
Schematic structures of a nematic mesophase and polymer–colloid complexes and polarized microscopy images of a conventional single–phase MBBA film and microfluidic MBBA microdroplets in the aqueous medium.

An important requirement of a liquid crystal material suitable for molecular diagnostic applications is its ability to form a mesophase at the standard temperature of 25 °C. On the other hand, a convenient research selection will be an LC material with well-characterized properties that may be helpful to explain interfacial phenomena in confinement. In this work, we used N-(4-methoxybenzylidene)-4-butylaniline (MBBA) liquid crystal (Figure 1, top left), represented by rod-like molecules, which form a nematic mesophase at room temperature (Figure 1, top right) and up to 45 °C [52].

For polymer and amphiphile aqueous solutes, we used sodium dodecyl sulfate (SDS) and poly(diallyl dimethylammonium chloride) (PDADMAC). These substances are an interacting pair of oppositely charged polyelectrolyte and surfactant. They can be considered as models of biological polymers and amphiphiles. Their association and phase behavior in macroscopic conditions and microfluidic confinement was characterized in our previous works [53,54], which can be helpful for analyzing their interactions with microfluidic MBBA dispersions, and the experimental results can be used for adapting a microfluidic LC environment to biopolymers such as nucleic acids or proteins. In addition, SDS is a proven surfactant used for producing a variety of LC dispersions for sensing applications [6,11,18]. SDS was successfully tested for producing MBBA microdroplets in our preliminary experiments (Figure 1, bottom right).

Dynamic light scattering (DLS) experiments and the literature data allowed to compare the molecular size characteristics of substances and dispersions, as shown in Figure 1. The characteristic sizes (radii of the equivalent spheres) of MBBA molecules and SDS ions evaluated from their molecular mobility data [55,56] are approximately 0.4 and 1 nm, respectively. Size characteristics of the PDADMAC SDS system were found to depend on the ratio of components. In a pure polymer solution, the hydrodynamic radius of PDADMAC macromolecules was found to be about 8–10 nm, according to the DLS results. If the molar concentration ratio of SDS ions to PDADMAC ionogenic groups (Equation (1))

(1) $$\beta =\frac{C_{SDS}}{C_{PDADMAC}}$$

was less than 0.4, the hydrodynamic radius of PDADMAC-SDS complexes did not exceed 50–70 nm. Further addition of SDS favored the formation of insoluble complexes due to the neutralization of polyelectrolyte charges [57]. Such complexes aggregated into sub-micrometer and micrometer range particles in a microfluidic channel, according to performed macroscopic and microfluidic experiments and DLS measurements. The diameters of the produced microfluidic MBBA dispersions varied in the range of dozens of micrometers.

MBBA, both in single phase and in dispersions, demonstrates a range of similar birefringence colors in polarized light (Figure 1, right), and therefore, shows a variety of molecular orientations with respect to the polarized light direction (perpendicular to the image plane).

Thus, microfluidic MBBA dispersions and the SDS-PDADMAC pair represent a flexible system with broad size characteristics, molecular mobility and phase behavior of its components. Such a system is potentially capable of demonstrating specific effects from interactions of its components on molecular and intermolecular levels.

### 2.2. Generation of MBBA Dispersions in Microfluidic Confinement

**Figure 2:**
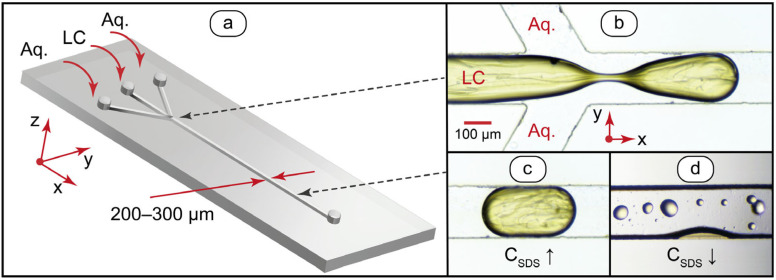
Design of the microfluidic chip and generation of disperse liquid crystal systems in a microchannel: (**a**) microfluidic flow-focusing device; (**b**) bright field optical microscopy image of MBBA microdroplet formation at 0.5 mm/s total flow velocity and the ratio of flow rates Q_Aq_/Q_MBBA_ = 5/1; (**c**) channel-wide MBBA microdroplet; (**d**) MBBA microdroplets immobilized on microchannel bottom surface. “Aq” is the aqueous phase represented by pure water or SDS and PDADMAC aqueous solutions infused on demand. Red arrows show the coordinate axes, the main channel size, and the inlets. Black dashed arrows show the channel points where microscopy images were taken.

A proven approach to generate multiphase systems in microfluidic confinement is infusing two or more immiscible liquids into multiple microchannel inlets, which merge into a single main channel [58]. Such devices were designed and fabricated for this work from polydimethylsiloxane (PDMS) (Figure 2), which is a standard material for laboratory-level fabrication of microfluidic chips. PDMS is optically transparent in the visible range and stable in a broad temperature range. For producing LC droplets, MBBA was infused into the central inlet, and aqueous solutions were infused into the side inlets (Figure 2a). Production of LC microdroplets was studied in our previous work based on a similar nematic LC [59], where we characterized an impact of aqueous and LC flowrates and their flow rate ratio on the orientation behavior of liquid crystal molecules. We selected and tested the flow velocities and the flow rate ratio for the MBBA aqueous SDS system according to a previous research on droplet generation of an analogous nematic LC system.

Experiments demonstrated that MBBA microdroplets were smoothly generated in a microchannel with surfactant concentration in the aqueous phase C_SDS_ > 5–6 mmol/L in the studied flow velocity range (Figure 2b,c). Lower concentrations of surfactant (C_SDS_ < 5 mmol/L) were suitable for droplet generation, but also resulted in the deposition of smaller size MBBA microdroplets on a microchannel surface, which broke up from the channel-wide droplets during their path downstream (Figure 2d). Such a mode allowed to generate 50–100 μm LC droplets immobilized on a microchannel surface. In pure water, droplet generation was unstable, which is in agreement with [58].

**Figure 3:**
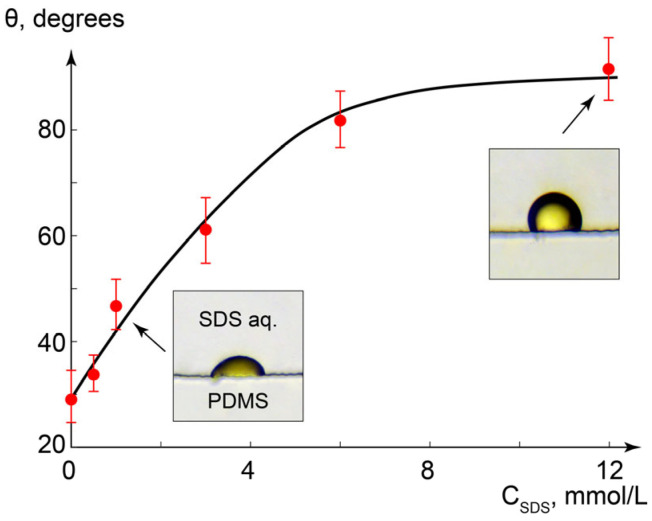
Impact of SDS concentration in the aqueous phase on contact angles of MBBA microdroplets on PDMS surface.

To evaluate the influence of surfactant concentration and microchannel walls on the behavior of confined MBBA dispersions, we measured contact angles of MBBA droplets immobilized on microchannel walls as well as on dry PDMS. Similar size microscale droplets (≈70–100 μm in diameter) were selected along the microchannel and analyzed to avoid a distorting effect on the contact angle from factors such as the droplet volume. Figure 3 summarizes the results.

Introduction of surfactant increases the contact angle from θ ≈ 30° (C_SDS_ = 0) to θ ≈ 90° (C_SDS_ > 6 mmol/L). The respective changes in the contact angle observed for the MBBA droplets deposited on a dry PDMS surface pre-processed with surfactant solutions were only from θ ≈ 35° to θ ≈ 55°. A more intensive increase in the contact angle found for MBBA-PDMS-SDS_Aq_ system can be caused by a specific contribution of the processes at the MBBA-liquid interface to the free energy reduction. In turn, this effect may indicate a gradual adsorption of SDS molecules on the surface of an MBBA droplet with an increase in SDS concentration until the surfactant molecules form a monolayer, which is in agreement with the literature [58].

Contact angle analysis and previous study results contributed to optimize the conditions for the generation of immobilized MBBA droplets in microchannels. The following algorithm was selected, unless otherwise specified: generating channel-wide droplets at the flow velocities U = 0.5–5 mm/s, at the concentration of the added surfactant C_SDS_ = 1 mmol/L. In such an SDS concentration, adhesion to a microchannel was high enough for sustainable formation of smaller droplets immobilized in the PDMS surface. To consider a possible impact of solution change on the geometry of pre-deposited LC droplets, their contact angles were re-evaluated after the infusion of a new aqueous solution of SDS or PDADMAC.

### 2.3. Characterizing Molecular Orientation Behavior of MBBA Dispersions in Microfluidic Aqueous Surfactant Media

**Figure 4:**
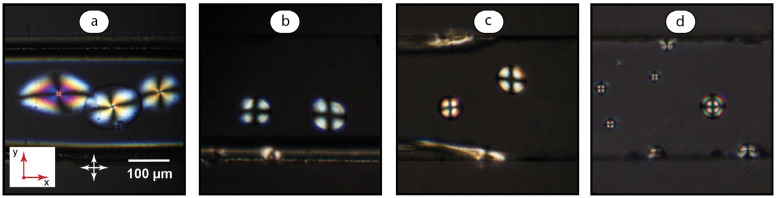
Polarized microscopy images of MBBA microdroplets in aqueous SDS solutions. SDS concentration, mmol/L: (**a**) 0; (**b**) 1; (**c**) 3; (**d**) 6. Red arrows represent coordinate axes with respect to Figure 2. Crossed arrows indicate the position of polarizers.

Liquid crystal dispersions in microfluidic channels represent anisotropic media with various ordering conditions. Transmission of polarized light through such media depends on the type of their molecular ordering. Microfluidic MBBA dispersions with different SDS concentrations in the aqueous phase were studied using polarized light microscopy. Figure 4 summarizes the results.

We analyzed similar size droplets (70–100 μm) at identical settings of polarized microscope and image acquisition software. Also, we analyzed tilt angles of LC molecules in central parts of the droplets, where the expected difference in thickness did not exceed several micrometers even with large contact angles. In such conditions, we can expect that the possible contribution of optical effects to the intensity and color of droplets can be similar and will allow to quantitatively distinguish the changes in molecular ordering of LC microdroplets upon the addition of surfactants and polymers to the aqueous phase.

Figure 4 demonstrates a distinct qualitative indication of surfactant additives. The color profiles of droplets in Figure 4b–d can be easily distinguished from each other, and from the LC droplets produced in pure water (Figure 4a). The pattern in Figure 4d is similar to those demonstrated in publications that describe biosensing capabilities of LC droplets [6,12,17,34], and is reported to be caused by the radial orientation of the LC molecules in the presence of surfactant.

The molecular orientation of liquid crystals can be defined from polarized microscopy images by comparing birefringence colors of LC films with those in the Michel–Levy chart. In this work, we used a method described in the literature [9]. According to this method, tilt angles of LC molecules can be calculated as follows (Equation (2):

(2) $$\Delta n_{eff}=\frac{n_{∥}n_{⟂}}{\sqrt{{n_{⟂}}^{2}{sin}^{2}\gamma +{n_{∥}}^{2}{cos}^{2}\gamma }}-n_{⟂}$$

where Δn_eff_ is birefringence, n_∥_ and n_⟂_ are anisotropic refraction indices, and γ is the tilt angle.

In this work, immobilized LC droplets were considered as variable thickness fragments of an LC layer. By approximating droplet shapes to spherical profiles, the thickness at every point can be calculated from the contact angle and the visible droplet diameter measured using optical microscopy tools. Droplet colors were compared with the Michel–Levy chart to calculate the tilt angles of LC molecules at various distances from the droplet centers. The calculation details are provided in Supplementary Materials.

**Figure 5:**
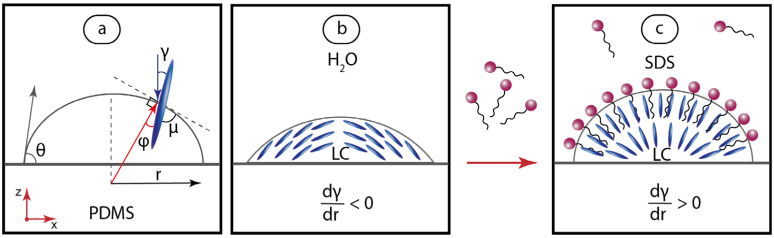
Schematic drawings (not to scale) representing suggested orientations of MBBA molecules in microdroplets on PDMS surface in aqueous SDS solutions: (**a**) geometry and characteristic angles; (**b**) C_SDS_ = 0; (**c**)—C_SDS_ > 1 mmol/L. Characteristic angles: θ—contact angle, γ—tilt angle, φ—angle to the radial director, μ—angle to the LC-water interface. Red arrows represent coordinate axes with respect to Figure 2.

Figure 5 demonstrates the droplet geometry with the respective suggested orientation of MBBA molecules in microdroplets.

Changes in the tilt angle γ (Figure 5a) with the distance from a droplet center dγ/dr are helpful for clarifying the alignment of LC molecules across a droplet. In water and in the C_SDS_ < 0.5 mmol/L solution, the tilt angle was evaluated to decrease from ~50° near the center to ~15° near the droplet edge (dγ/dr < 0). An opposite molecular alignment of LC molecules was observed for droplets in >3 mmol/L SDS: the evaluated tilt angle approached 0° near droplet centers. Figure 5b,c demonstrate the respective suggested molecular ordering structures of LC microdroplets in pure water and surfactant solution.

To quantify molecular orientation in LC materials, an order parameter is defined. For the director perpendicular to the LC-water interface, the order parameter is given in Equation (3):

(3) $$S_{order}=\frac{3{cos}^{2}\varphi -1}{2}$$

where φ is the angle between the director (Figure 5a, red arrow) and the long axis of LC molecules. For an ordered mesophase, S → 1; for an isotropic liquid S → 0.

The characteristic size of SDS molecules is several orders of magnitude lower that the sizes of LC microdroplets. Therefore, assuming that SDS-initiated LC anchoring is uniform along the interface between the LC and aqueous phases, the order parameter S and the angle μ (Figure 5a) were supposed to be generally independent on the distance from the droplet center. These parameters were calculated from the tilt angles at 8–10 points, selected at different distances from the droplet center. Average values of LC molecules to the LC-water interface were then calculated with the respective deviations from the average. To exclude a possible impact of PDMS surface anchoring, these points were selected in central parts of the droplets (within 2/3 of their radius). Also, at such a distance from the droplet center, differences in its thickness did not exceed several micrometers, and we could simplify calculations using the droplet thickness evaluated for its center. The calculation details are provided in Supplementary Materials.

The values of S and μ were found to be similar for the studied droplet points. These parameters can therefore be more convenient for quantifying the ordering of LC molecules in droplets than tilt angles. Table 1 summarizes the average S and μ values.

According to Table 1, MBBA droplets in pure water demonstrate the highest deviation in their molecular orientation from the radial director and the respective lowest order parameter. Introduction of surfactant to the aqueous phase was found to increase both the interface angle and the order parameter. At C_SDS_ > 3 mmol/L, the orientation of LC molecules is nearly homeotropic (μ → 90°), and the order parameter is the highest (S → 1).

Thus, the impact of surfactant concentration on the orientation of LC molecules in immobilized microfluidic droplets can be evaluated with the visual analysis of droplet color patterns in polarized light and also quantified with their birefringence analysis. The next stage of this work is focused on analyzing the impact of polymer additives to the LC molecular ordering in MBBA-SDS dispersions.

### 2.4. Characterizing Molecular Orientation Behavior of MBBA Dispersions in Microfluidic Polymer–Surfactant Media

Performing interactions of surfactants and polymers is a sustainable, fundamental and applied approach to create organized and responsive colloid media. Not limited to synthetic polymers and amphiphiles, vital biopolymers such as nucleic acids and proteins associate with surfactant molecules and undergo a variety of nanoscale structural transformations [57].

**Figure 6:**
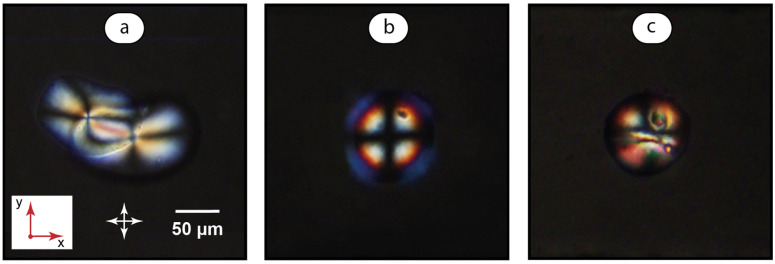
Polarized microscopy images of pre-deposited MBBA microdroplets. Further infusion of aqueous phases: (**a**) PDADMAC 1 mg/mL; (**b**) PDADMAC 1 mg/mL after SDS 1 mmol/L; (**c**) SDS 3 mmol/L after PDADMAC 1 mg/mL. Red arrows represent coordinate axes with respect to Figure 2. Crossed arrows indicate the position of polarizers.

On a molecular level, binding surfactant molecules to polymers may result in their concentration in a polymer matrix even if the total surfactant concentration in solutions is 1–2 orders of magnitude below its critical micellization concentration [57]. Introduction of polymer macromolecules to a LC-surfactant dispersion may therefore exert a significant impact on the LC molecular alignment. Hence, at this stage of the work, we studied the effect of PDADMAC additives on the behavior of microfluidic MBBA droplets in SDS solutions. Figure 6 summarizes the results.

Figure 6a demonstrates the reference experiment with no surfactant added. The aqueous polymer solution was infused into the main channel after creating a LC dispersion in water. The color pattern of droplets in Figure 6a is similar to that in Figure 4a captured for LC droplets in pure water. Same patterns were observed after polymer injections in the studied concentration range of 0.01–1 mg/mL. Such results agree with the fact of lower surface activity of well-soluble polymer macromolecules and with the expected weaker orienting effect on LC as compared to surfactant ions.

In the next experiments (Figure 6b,c), the LC droplets were pre-deposited in 1 mmol/L SDS solution. Figure 6b shows the images after LC and SDS infusion was stopped and PDADMAC solution was infused through the lower side input. The resulting birefringence pattern differs from that created by the individual surfactant solutions of 1 mmol/L (Figure 4b). Its variety of alternating colors resembles the pattern developed at higher surfactant concentrations (Figure 4d). Similar effects were observed for polymer injections in the studied concentration range of 0.01–1 mg/mL.

Properties of polymer–surfactant systems depend on the polymer–surfactant concentration ratio (Equation (1)). In Figure 6b, we can see the results of PDADMAC interactions with the residual SDS in a microchannel that occur at low values of the SDS/PDADMAC ratio β (Equation (1)). To test the behavior of the system at higher β, SDS was re-infused into the main channel. Figure 6c shows the resulting transformation of the droplet color profile into a more random pattern with a variety of birefringence colors, and therefore, the orientations of LC molecules.

To quantify the ordering of LC molecules in the presence of PDADMAC, their angles to the LC-water interface and the order parameters were calculated. Table 2 summarizes the results.

We can see in Table 2 that the radial order parameter is relatively low for polymer-only additives. Infusion of PDADMAC solutions into surfactant-mediated MBBA dispersions increases the order parameters and angles μ to the LC-Aq interface up to the values calculated for much higher surfactant concentrations (6 mmol/L and above). The μ and S values calculated for the droplet in Figure 6c are again much lower than those found for an ordered radial orientation of the LC molecules.

It can be useful to discuss the experimental results obtained for polymer–surfactant mixtures in the context of the underlying molecular mechanisms governing the alignment of nematic crystals when interacting with polymers and colloids. Anchoring of nematic liquid crystals to various surfaces can be precisely controlled via modifying these surfaces with substances that favor homeotropic or planar alignment, delicate mechanical treatment of these surfaces and also via applying the temperature factor, which is also a governing parameter for intermolecular interactions and alignment of LC molecules [60,61].

LC droplets in pure water demonstrate neither homeotropic nor planar alignment (the average angle to the LC-water interface is about 40–45 degrees). We can expect intermolecular interactions between LC molecules on the droplet surface and water molecules at the interface. In [60], various molecular mechanisms are reported to be potentially responsible for LC alignment at a surface, namely dispersion forces and hydrogen bonding as well as steric interactions between LC molecules at the interface. A combination of these mechanisms may be responsible for LC molecules forming such an inclined orientation. Therefore, on one hand, LC molecules tend to incline towards the contacting aqueous surface, which assumes a certain surface interaction of LC rods with the interface. On the other hand, a combination of intermolecular forces at the interface is not sufficient for a perfectly planar alignment in the studied experimental conditions.

**Figure 7:**
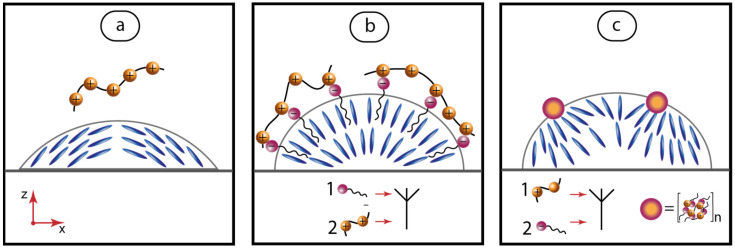
Schematic drawings (not to scale) representing suggested orientations of MBBA molecules in microdroplets on PDMS surface in aqueous PDADMAC and SDS solutions. Concentrations and infusion sequence: (**a**) PDADMAC 1 mg/mL; (**b**) PDADMAC 1 mg/mL after SDS 1 mmol/L; (**c**) SDS 3 mmol/L after PDADMAC 1 mg/mL. Red arrows represent coordinate axes with respect to Figure 2.

The proposed alignment of LC molecules in the presence of polymer–surfactant solutions is shown in Figure 7. Addition of polymer macromolecules introduces another molecular interaction mechanism to favor the alignment of LC molecules along the aqueous interface, which is the interaction of LC rods with charged molecular groups [60] on the polymer chain and polymer backbone. This factor may also be responsible for low values of molecular tilt angles with respect to the LC-aqueous interface (Figure 7a).

Introduction of surfactant to the aqueous phase adds a substance, which initiates a competing homeotropic alignment of the LC molecules. In this case, the underlying molecular mechanisms may be the intermolecular interactions between the LC molecules and alkyl chains of surfactant molecules, which tend to take place inside the LC droplets. An increase in SDS concentration is supposed to enhance the homeotropic anchoring effect and demonstrate a continuous change in the tilt angle to homeotropic. Such an effect was demonstrated in [61] for a system of two substances, which initiated both homeotropic and planar anchoring.

An orientational behavior of LC molecules in the presence of PDADMAC can be explained by the electrostatic binding of surfactant ions to PDADMAC charged groups, resulting in the stabilizing effect of a polymer matrix on surfactant molecules adsorbed on LC droplets, and the resulting enhancement of homeotropic anchoring at low surfactant concentrations (Figure 7b).

According to our preliminary experiments, soluble nanoscale complexes are supposed to form at low SDS/PDADMAC ratio β. Nanoscale sizes of such complexes assumes microscopically uniform conditions at LC droplets with a polymer–surfactant layer, and the resulting uniform homeotropic orientation of the LC molecules and the maximized order parameter. Figure 6b and Figure 7b illustrate a synergistic effect of surfactant and polymer additives on molecular ordering in LC dispersions.

At high β resulting, from an additional SDS infusion, insoluble complexes are supposed to form. These complexes aggregate further into macroscopic particles and precipitate in microchannels. Such an effect was observed and analyzed in our previous microfluidic experiments with polymer–surfactant solutions [53,54], and also confirmed in this work for a consecutive infusion of SDS and PDADMAC solutions into a microfluidic channel. Adsorption of such microparticles on LC droplets is supposed to be responsible for a non-uniform orientation of LC molecules across the droplet (Figure 7c). Figure 6c and Figure 7c illustrate the molecular orientation response of LC dispersions to a fundamental phenomenon in colloid chemistry and biochemistry that is a coagulation of insoluble particles.

It is important to note that given the dynamic nature of polymer complexes, even slight temperature fluctuations can trigger alterations in their molecular arrangement. Consequently, it will affect liquid crystal molecule orientation. The presence of polymer and colloid solutions in the contacting aqueous phase is also expected to impact the transition temperature of a liquid crystal.

The experiments therefore were conducted at the standard temperature of 25 °C. In addition, we conducted preliminary experiments to study the effect of temperature on the transitions of microfluidic single-phase LC systems and LC dispersions in the presence of aqueous surfactant solutions. At 25 °C, all the studied systems demonstrated mesophase states, clearly distinguishable by the birefringence patterns in polarized microscopy images. The mesophase structure was observed at higher temperatures until it approached the MBBA transition temperature reported in the literature.

An accurate measurement of the MBBA transition temperature in microfluidic channels in the presence of polymer and surfactant aqueous solutions will require consideration of the fact that liquid crystal droplets are not in direct contact with the heating element, and are heated through microchannel walls via a PDMS layer. Studies on more precise temperature are planned for the future. Within the framework of this paper, however, we demonstrated that a possible impact of polymer and colloid additives on the transition temperature of microfluidic LC dispersions was not so considerable to affect the transition behavior of the nematic mesophase at the standard temperature of the performed experiments.

### 2.5. Impact of PDADMAC Diffusion on the Behavior of MBBA Dispersions in Confined Microflows

Microfluidic chips for molecular diagnostics and sensing and are designed for dynamic flow conditions [33,48]. Typical operation conditions of such devices are represented by multiple laminar flows of biochemical species and their non-uniform concentrations across and along microchannels.

**Figure 8:**
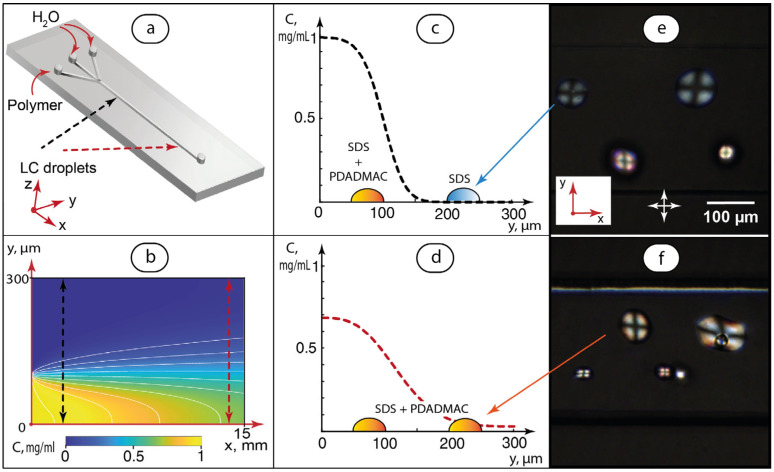
Modeling PDADMAC diffusion in a microchip and experimental verification of its impact on molecular orientation of MBBA in microdroplets pre-deposited across the main channel: (**a**) infusion of PDADMAC solution (Q = 0.5 μL/min) and H_2_O (total Q = 1 μL/min), dashed arrows represent the location of LC droplets used for the analysis; (**b**) PDADMAC concentration distribution in the entire channel at height h = 15 μm from the lower PDMS surface; (**c**,**d**) PDADMAC concentration distributions at microchannel cross-sections; (**e**,**f**) respective polarized microscopy images of MBBA microdroplets. Solid red arrows show the coordinate axes and the inlets. Dashed arrows show the channel points for which cross-sectional polymer concentrations were calculated.

The final stage of this work, therefore, focused on testing the molecular orientation behavior of LC dispersions in concentration gradient conditions. For the experiment, we selected PDADMAC and infused it into SDS-mediated MBBA dispersion, as shown in Figure 4b. Experimental design, numerical simulations of the PDADMAC concentration field, and the respective polarized microscopy images are shown in Figure 8.

In this experiment, polymer solution was infused through the lower side input (Figure 8a). Water was infused through other two inputs. The flow rate of PDADMAC was set as 1/3 of the total flow rate, so its flow occupied 1/3 of the main channel after the junction. The Reynolds number calculated for such flows in the main channel was Re << 1. The flow was, therefore, strongly laminar, so the transverse mass transfer of PDADMAC macromolecules was supposed to occur via diffusion only.

In stationary laminar flow conditions, the concentration distribution of the solute in a microchannel can be described using the following convection–diffusion equation:

(4) $$U\frac{\partial C_{P}}{\partial x}=D_{P}\frac{\partial^{2}C_{P}}{\partial y^{2}}$$

where U(y) is the flow velocity, C_P_ is the PDADMAC concentration, D_P_ is the diffusion coefficient of PDADMAC macromolecules according to the DLS data, and x and y are the coordinates according to Figure 8a.

Equation (4) was solved using Matlab with the boundary conditions set using the microchip geometry and infusion of fluids. The details are provided in Supplementary Materials.

Figure 8b shows the solution of Equation (4) for the entire main channel with white iso-concentration curves. It visualizes a gradual diffusion of PDADMAC macromolecules across the channel as they proceed downstream. Polymer concentrations were then extracted from the general solution for the cross-sections marked by black and red dashed arrows. Figure 8c,d demonstrate the concentrations plots. Finally, Figure 8e,f show the images of the LC droplets captured in the main channel areas marked by the dashed arrows in Figure 8b.

In Figure 8e, we can see distinct differences in the color patterns of the droplets in the beginning of the main channel. The lower droplets were in the PDADMAC flow and exhibited a different birefringence profile. The calculated concentration of polymer was nearly zero in the area occupied by the upper droplets, so their color pattern remained unchanged and similar to that shown in Figure 4b.

Near the main channel end (Figure 8f), all the droplets obtained a birefringence profile that was previously observed for a close-to-radial orientation of the LC molecules. Such an optical behavior agrees with the simulation results predicting that a certain fraction of PDADMAC macromolecules diffused across the entire main channel near its end in such flow conditions.

With an increase in the PDADMAC/H_2_O flow rate ratios or decrease in the total flow rate, the image shown in Figure 8e was observed closer to the junction of the inlet channels that agreed with the respective numerical simulations of PDADMAC concentration distribution.

Therefore, the experiment shown in Figure 8 demonstrated that orientations of LC molecules in immobilized droplets can be sensitive to non-uniform concentrations of dissolved species in convection–diffusion conditions of dynamic microflows.

## 3. Discussion

The experimental results of this work highlight a remarkable feature of liquid crystals, that is, their ability to undergo ordering by interacting with biologically important molecular agents represented by polymers and amphiphiles. In turn, these molecular level effects were found to be directly associated with transmitted light characteristics of LC dispersions. Changes in such properties are easily detectable using polarized optical microscopy tools.

In this paper, we focused on analyzing the molecular behavior of microfluidic nematic dispersions represented by 70–100 μm droplets immobilized on microchannel surfaces. Such dispersions offer certain advantages over microfluidic systems represented by LC films or confined mobile LC microdroplets in aqueous flows, which are predominantly discussed in previous research publications found after the literature review. Firstly, they require no additional microchannel modification and can be easily produced via conventional flow-focusing devices by maintaining a sufficient LC adhesion to a PDMS surface. Secondly, immobilized droplets allow to analyze the orientation of LC molecules in dynamic flows and mass transfer conditions. Thirdly, immobilized LC droplets can be approximated as variable thickness fragments of mesophase films on a PDMS surface. Comparing birefringence colors in such droplets with those in a Michel–Levy chart turned out to be applicable in assessing the tilt angles of LC molecules in such droplets, and therefore, allowed to quantify their molecular ordering in various conditions.

SDS additives were demonstrated to initiate homeotropic ordering of MBBA molecules at the LC-water interface. Although this effect was well-proven in previous papers describing similar LC-surfactant microdroplet systems, we proposed to quantify the aligning impact of surfactant molecules on LC droplets by introducing the order parameter, which is typically used for continuous LC films. In this case, the molecular orientation director is perpendicular to the curved LC-water interface.

Surfactants are known to intensively interact with polymers, especially with polyelectrolytes. Binding surfactant molecules using a polymer matrix at small surfactant/polymer ratios is supposed to create a more stable polymer–colloid surface layer on the surface of an LC droplet at low surfactant concentrations, and therefore result in the observed increase in the order parameter of LC molecules calculated from the polarized microscopy data. It makes surfactants convenient intermediates to adapt LC dispersions for sensing polymer macromolecules, such as biopolymers in microfluidic devices designed for molecular diagnostics.

On the other hand, the size of the insoluble microscopic PDADMAC-SDS coagulates emerging at high surfactant-to-polymer molar ratios is comparable to the diameters of LC dispersed phase particles. Such coagulates randomize molecular orientation in LC microdroplets, reduce the LC order parameter, and produce non-symmetrical polarized microscopy images. Such sensitivity of LC droplets to microparticles is also promising for developing microfluidic sensing platforms that can detect coagulation in microflows.

Highly ordered laminar flows in microfluidic confinement offer a diffusion-governed molecular transport and predictable concentration fields of dissolved substances. The observed sensitivity of surfactant-modified MBBA droplets to variable polymer concentrations across a microchannel is potentially suitable for on-chip characterization of mass transfer processes or chemical reactions.

Despite their application potential in microfluidic sensing, the molecular orientation effects in polymer–colloid LC dispersions were demonstrated in this paper using a model polymer–surfactant system. It should also be noted that SDS is a toxic substance and further biomedically oriented experiments will require the use of more biologically friendly surfactants. Therefore, for a vital practical application of microfluidics, such as biomedicine, these research results should be verified and adapted to biochemical species represented by biopolymers and biological colloids. Further research plans will therefore focus on studying molecular orientation of confined nematic microdroplets in surfactant-mediated flows of biopolymers, such as chitosan, which is a common component of drug delivery systems. We will also consider an idea to use phase contrast microscopy for analyzing the optical behavior of LC microdroplets in the future. Another future research focus will include a comprehensive analysis of the impact of temperature on molecular orientations of LC molecules and transitions of nematic mesophase in various surfactant and polymer concentrations in aqueous flows.

## 4. Materials and Methods

### 4.1. Materials and Solutions

Microfluidic devices were fabricated from polydimethylsiloxane (PDMS) Sylgard^TM^ 184 silicone elastomer (Dow Corning Midland, MI, USA) and used as received. It came as a two-part elastomer kit (the pre-polymer and curing agent). SU-8 3050 photoresist (Microchem Corp., Westborough, MA, USA) was used to produce a mold for microfluidic chips.

For the liquid crystal phase, the nematic liquid crystal N-(4-methoxybenzylidene)-4-butylaniline (MBBA) was used. It was purchased from Reachem, Moscow, Russia, and used as received. It exhibits liquid crystal properties at the standard 25 °C temperature, at which all the polarizing microscopy observations were performed.

Polydiallyldimethylammonium chloride (PDADMAC) was purchased from Sigma Aldrich, St. Louis, MO, USA. The polymer is sold as a viscous liquid (20% aqueous solution) and it was used as received. Sodium dodecyl sulphate (SDS) was purchased from BDH Limited, Poole, England and used as received. Surfactant is sold as a powder.

Bidistilled water was used for the aqueous phase. Before performing microfluidic experiments, the solvent was passed through 0.45 µm Millipore polytetrafluoroethylene (PTFE) filters, purchased from Merck, Darmstadt, Germany.

For microfluidic experiments, bulk solutions of 1 mg/mL PDADMAC were produced from the commercial sample and allowed to dissolve overnight. Such a concentration corresponds to the range of polyelectrolyte swelling, and is expected to provide intensive polymer–surfactant interactions in solutions that was studied in our previous work [54]. The respective concentration of monomers in 1 g/L PDADMAC solution was ~6 × 10^−3^ mol/L. Bulk solutions of 6 × 10^−3^ mol/L SDS were produced by dissolving dry surfactant in deionized water to provide the same molar concentration of surfactant ions with respect to polymer ionogenic groups. Lower concentrations of PDADMAC and SDS solutions were produced on demand by dissolving their initial solutions with filtered bidistilled water. In the studied concentration range, the viscosity of the polymer and surfactant solutions was not considerably different from that of a pure solvent and did not affect smooth infusion of the solutions through microfluidic chip inlets.

### 4.2. Methods

#### 4.2.1. Characterizing Properties of MBBA and PDADMAC-SDS Colloid Systems in Bulk and Microfluidic Confinement

Preliminary characterization of LC microdroplets in microfluidic confinement was performed via digital optical microscopy using a Levenhuk D320 optical microscope (Levenhuk, Tampa, FL, USA). Microscopy images were captured at 100× magnification using a ToupCam E3ISPM08300KPB camera (Touptek, Hangzhou, China).

Orientation behavior of the LC dispersed media in microfluidic flows were studied via polarized optical microscopy (POM) using an Olympus BX51 microscope (Olympus, Tokyo, Japan), equipped with a high-precision Linkam heating system that allows to provide uniform temperature conditions for experiments. Microscopy images were captured at 100× and 500× magnification using a ToupCam E3ISPM08300KPC camera (Touptek, Hangzhou, China).

The microscopy images were obtained via the ToupView camera software, version 4.11 provided with the microscope cameras. For further comparison of the droplet color profiles, all the microscopy images were taken at identical settings of both the microscopes and the ToupView software supplied with the cameras. The color profiles of the droplets were compared with the modified Michel–Levy chart, which considers the impact of image file color spaces and formats [62].

Processing of microscopy images, evaluation of the molecular ordering characteristics of MBBA in microdroplets, and modeling of the PDADMAC diffusion in a microchannel was performed via Matlab software, version 2021b with the Partial Differential Equations Toolbox. In Table 1 and Table 2, each entry is calculated based on the dataset of 8–10 points selected along the surface of liquid crystal droplets. The statistical processing of the obtained data was then performed to calculate the average values and deviations.

The images of microdroplets, shown in Figure 4 and Figure 8, represent different size microdroplets, which were immobilized on microchannel walls. We decided to show the images of multiple droplets in Figure 4 and Figure 8 because they demonstrate a real microchannel environment and visible qualitative differences in color profiles of the droplets upon addition of surfactant and polymer solutions. The representative images were slightly formatted for better visualization.

For the quantitative analysis and data summarized in Table 1 and Table 2, however, we analyzed only images of similar individual microdroplets size (regular round shapes and 70–100 μm in diameter with the average diameter of droplets about 80 μm). Such droplets were the most convenient for analysis because of sufficient size and related quality of microscopy images as well as distinguishable birefringence profiles.

We were also careful with the intensity and settings of microscopy images. All the images for the quantitative analysis were taken at identical settings of the microscope and the capturing software.

Contact angles were measured with the Kruss DSA20 Easy Drop system. For evaluating contact angles of MBBA droplets on a dry PDMS surface, droplet images were captured via the Kruss Easy Drop camera. For measuring contact angles of MBBA droplets dispersed in microfluidic aqueous media, droplet images were captured and saved for further processing using the respective optical microscope camera software. To avoid a possible impact of droplet volume in the contact angle experiments, droplets with similar sizes were analyzed. After capturing, all the images were processed via the Kruss DSA20 Easy Drop software, version V1-03 to evaluate the contact angles.

Hydrodynamic diameters of PDADMAC macromolecules and PDADMAC-SDS complexes were measured using a Malvern Zetasizer Nano ZS light scattering system. All the DLS measurements were repeated at least three times to obtain reproducible results. The reported diameters of particles measured via DLS correspond to the maximums of the number-average distribution curves provided using Malvern Zetasizer Nano ZS version 7.13 software reports.

#### 4.2.2. Fabricating Microfluidic Devices and Preparing the Experimental Setup

Microfluidic devices were fabricated using standard photolithography techniques [63]. The chips with rectangular microchannels were produced using this technology. The length, width, and height of all the microchannels were 15 mm, 300 μm, and 100 μm, respectively. SU-8 photoresist and a transparent photomask with a negative image of a microchip were used to produce a 100 µm thick mold of microfluidic chips on top of a 3 inch silicon wafer. PDMS pre-polymer was mixed with a curing agent, poured over the mold, and allowed to cure in an over for 4 h in 60 °C. Once cured, PDMS was peeled off the mold and bonded to a flat PDMS slab via 1 min plasma treatment using the Harrick Plasma Cleaner PDC-23G, Ithaca, NY, USA. The PDMS device was then heated in an oven at 180 °C for 1 h to complete the bonding of the two polymer layers.

The LC and aqueous phases were infused into microfluidic devices using Shenchen ISPLab01 syringe pumps (Baoding Shenchen Precision Pump Co., Ltd., Baoding city, China). In this work, the flow velocities of the LC phase and aqueous phases varied in the range up to 0.5–5 mm/s. To provide the same hydraulic paths for all the fluids to all the inlets, PTFE tubes of identical lengths (10 cm) and internal diameters that fit the same needle tips inserted into microchip outputs (20 G-type needles, 0.9 mm diameter) were used. These tubes were connected to identical 1 mL syringes installed into the syringe pumps.

## 5. Conclusions

Microfluidic MBBA dispersions offer a range of quantitatively tunable molecular ordering options in the presence of polymer–surfactant colloids. Infusion of SDS solutions increased the LC microdroplet contact angles and the respective homeotropic molecular order parameter from appr. S ≈ 0.3 (C_SDS_ < 0.5 mmol/L) to S → 1 (C_SDS_ > 3 mmol/L). Infusion of PDADMAC after SDS (<1 mmol/L) also enhanced the order parameter to S → 1. Such effects indicate the formation of homeotropic MBBA composites with adsorbed SDS molecules or PDADMAC-SDS colloids. Additional infusion of SDS after PDADMAC randomized orientation of the LC molecules in the droplets on a microscopic level due to effect of insoluble polymer–colloid microparticles. In parallel to PDADMAC-SDS and water flows, LC microdroplets with different molecular orientations were found to coexist in predictable conditions of polymer diffusion.

The discussed molecular behavior of the studied LC-surfactant-polymer systems offers new options for applications of microfluidic LC dispersions to in-flow analysis of polymers and amphiphiles or functional sensing in lab-on-chip platforms.

## Figures and Tables

**Table 1:** Average parameters of MBBA molecular ordering in microdroplets on PDMS surface: impact of SDS concentration.

C_SDS_ × 10^3^, mol/L	Angle μ to LC-Aq Interface	Order Parameter S
0	45±3	0.24 ± 0.06
0.5	56 ± 4	0.52 ± 0.11
1	71 ± 4	0.83 ± 0.07
3	82 ± 6	0.96 ± 0.04
6	87 ± 2	0.99 ± 0.01
12	88 ± 2	0.98 ± 0.01

**Table 2:** Average parameters of MBBA molecular orientation in microdroplets on PDMS surface: impact of PDADMAC additives.

C_PDADMAC_, mg/mL	Infusion ^1^	Angle μ to LC-Aq Interface	Order Parameter S
1	P only	39 ± 15	0.26 ± 0.11
0.01	P → S	79 ± 9	0.92 ± 0.10
0.1	P → S	86 ± 3	0.98 ± 0.02
1	P → S	87 ± 2	0.99 ± 0.01
1	S → P → S	47 ± 5	0.29 ± 0.12

^1^ Polymer (P) and surfactant (S) solutions were infused into the microchannel with pre-deposited MBBA microdroplets.

## Data Availability

Not applicable.

## Supplementary Materials

The following supporting information can be downloaded at: https://www.mdpi.com/article/10.3390/ijms241713555/s1.
