# Supplementary material for: Tuning Molecular Orientation Responses of Microfluidic Liquid Crystal Dispersions to Colloid and Polymer Flows
Source: Int J Mol Sci. 2023 Aug 31;24(17):13555. doi: 10.3390/ijms241713555 (PMC10488184; doi:10.3390/ijms241713555)
Supplement: Supplementary file 1 [file ijms-24-13555-s001.zip › ijms-2579558-supplementary.pdf]

Calculating the LC Droplet Thickness

To calculate the birefringence by using Michel-Levy Chart, one should now the thickness of the LC phase. Assuming that the profile of a droplet is close to spherical, this parameter can be calculated from the contact angle θ and the radius of the immobilized droplet b (Fig. SI1).

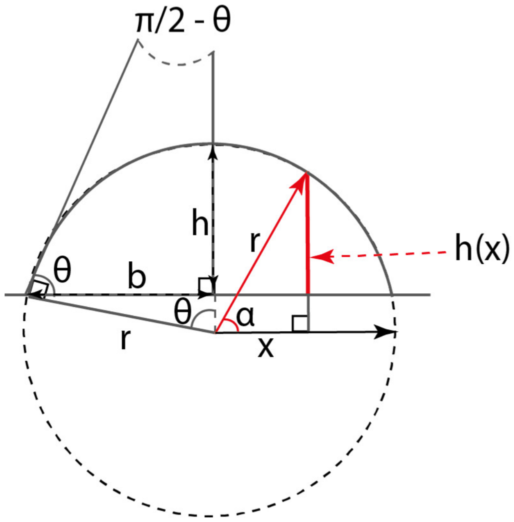

Figure SI1. LC droplet geometry. Characteristic lengths, and angles.

Let us calculate the thickness of the droplet in the center from the contact angle and the visible radius b:

$$\sin\theta = \frac{b}{r} \quad (1)$$

then:

$$r = \frac{b}{\sin\theta} \quad (2)$$

also:

$$\cos\theta = \frac{r-h}{r} \quad (3)$$

then:

$$h = (1 - \cos\theta)r \quad (4)$$

and the droplet thickness can be calculated as follows:

$$h = \frac{b(1-\cos\theta)}{\sin\theta} \quad (5)$$

Proceed to calculating droplet thickness at any point at the distance x from the droplet center:

Introduce the dimensionless parameter t :

$$t = \frac{x}{b} = \frac{x}{r\sin\theta} \tag{6}$$

Therefore:

$$x = tr\sin\theta \tag{7}$$

Trigonometric functions of the angle α:

$$\cos\alpha = \frac{x}{r} = t\sin\theta \tag{8}$$

and

$$\sin\alpha = \frac{h(x)+r-h}{r} = \frac{h(x)}{r} + \cos\theta \tag{9}$$

Consider that:

$$\sin^2\alpha + \cos^2\alpha = 1 \tag{10}$$

then:

$$t^2\sin^2\theta + (\frac{h(x)}{r} + \cos\theta)^2 = 1 \tag{11}$$

Continue transformations:

$$\frac{h(x)}{r} + \cos\theta = \sqrt{1 - t^2\sin^2\theta} \tag{12}$$

then:

$$h(x) = r(\sqrt{1 - t^2\sin^2\theta} - \cos\theta) \tag{13}$$

and finally:

$$h(x) = \frac{b}{\sin\theta}(\sqrt{1 - t^2\sin^2\theta} - \cos\theta) \tag{14}$$

Equation (14) was used for calculating droplet thickness at different points of analysis from the contact angle and the visible diameter of immobilized droplets. It assumes that the profile of droplets was close to spherical, that agreed with microfluidic

images if droplet profiles on microchannel side walls. The difference in droplet thickness in the analysis points was not, however, more than 3-4 μm. This clarification was, nevertheless, helpful for performing a more accurate analysis of birefringence colors with Michel-Levy chart.

Calculating the Tilt Angle and the Order Parameter

The values of birefringence for different points at LC droplets were calculated for the respective LC phase thicknesses at different points of droplets by comparing their colors with those on the Michel-Levy chart [1] considering image color corrections [2].

With the polarizers crossed as shown in Fig. 4 of the manuscript, the birefringence profiles of the droplets are represented by dark crosses symmetrical to "x" and "y" axes of the microchip. Other droplet sections demonstrate a distinguishable pattern of colors. We selected 8-10 points in the central parts of droplets (within 2/3 of their diameter, where the difference in thickness does not exceed several micrometers even for droplets with large contact angles) for color analysis and estimated their birefringence.

To transform the birefringence values into the tilt angles, the following equation was used:

$$\Delta n_{eff} = \frac{n_{\parallel} n_{\perp}}{\sqrt{n_{\perp}^2 \sin^2\gamma + n_{\parallel}^2 \cos^2\gamma}} - n_{\perp} \tag{15}$$

where Δn_{eff} is birefringence, $n\|$ and $n\perp$ are anisotropic refraction indices, γ is the tilt angle.

The tilt angle γ can be derived from Eq. 15 as follows:

$$\gamma = \arccos\left(\sqrt{\left(\frac{n_{\parallel} n_{\perp}}{\Delta n_{eff} + n_{\perp}}\right)^2 - n_{\perp}^2}\,\frac{1}{\sqrt{n_{\parallel}^2 - n_{\perp}^2}}\right) \tag{16}$$

The refraction indices of MBBA were evaluated from [3]. We used the LBD daylight filter in the Olympus BX51 microscope, which narrowed the light from the illuminating lamp closer to the yellow color area. and used average values of the refraction indices for the λ = 500-600 nm, which did not change considerably in this range so as the resulting values of tilt angles and order parameters.

The angle φ between a liquid crystal molecule and the radial director (Fig. SI2, red arrow) can be calculated from the angle α (Eq. 8) and the tilt angle γ (Eq. 16).

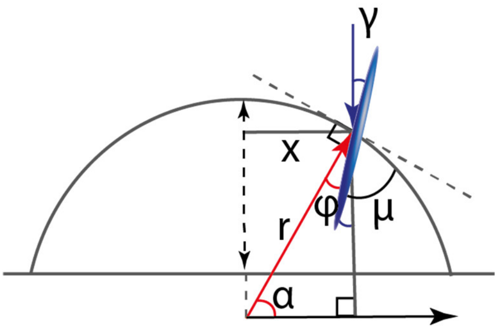

Figure SI2. The angle φ between a liquid crystal molecule and the radial director from the droplet geometry.

The resulting order parameter S was calculated at different points of LC droplets from the tilt angle as follows:

$$S_{\mathrm{order}} = \frac{3\cos^2\varphi - 1}{2} \tag{17}$$

and the angle μ between the liquid crystal molecule and the LC-Aq interface, which is convenient for characterizing the radial orientation of the LC molecules, is calculated as follows:

$$\mu = \frac{\pi}{2} - \varphi \tag{18}$$

Modeling Polymer Diffusion in a Microfluidic Channel

The model was developed for two contacting flows: polymer solution and the solvent. Fig. SI3 demonstrates the respective geometry.

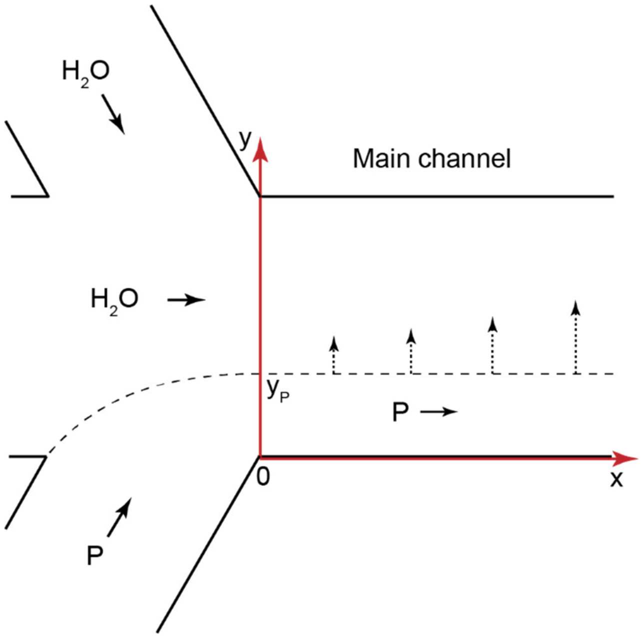

Figure SI3. Flow arrangement in a flow-focusing chip for the transverse polymer diffusion.

For the stationary conditions of microfluidic flow and predominant transverse diffusion of dissolved species, polymer concentration field can be described by the following convection-diffusion equation:

$$U\frac{\partial C_P}{\partial x} = D_P\frac{\partial^2 C_P}{\partial y^2} \tag{19}$$

where U(y) is the flow velocity, C_P is the PDADMAC concentration, D_P is the diffusion coefficient of PDADMAC macromolecules according to the DLS data, x and y are the coordinates.

The flow velocity U distribution in a microchannel can be approximated by the following equation:

$$U(y, z) = \beta(y, z)U \tag{20}$$

Here U = Q/(WH) is the flow velocity calculated from the flowrate, and β is the function describing the parabolic flow velocity distribution in a microchannel:

$$\beta = \frac{9}{4}[1 - (2\frac{y}{W} - 1)^2[1 - (2\frac{z}{H} - 1)^2 \tag{21}$$

where W is the microchannel width, H is the microchannel height, z is the vertical coordinate.

In this work, Eq. 19 was solved with the flow velocity set at specific height over microchannel bottom surface z = 15 μm that is an average height of immobilized LC droplets in the experiments. It allowed to simplify solution results and represent them as cross-sectional 2D plots.

The boundary conditions for the junction of the flows were introduced according to the microchannel geometry shown in Fig. SI3:

$$C_P(x = 0, y) = \begin{cases} C_P^0, y \geq y_P \\ 0, y < y_P \end{cases} \quad (22)$$

where y_P is the transverse coordinate of the polymer flow boundary that is calculated from the flowrate ratio of polymer solution and the aqueous phase [4]:

$$y_P = W\frac{Q_P}{Q_P + Q_{H2O}} \quad (23)$$

the boundary conditions for the microchannel walls are introducing assuming zero flow of dissolved species through the walls [3]:

$$\frac{\partial C_P}{\partial y}_{(0,W)} = 0 \quad (24)$$

Due to intrinsic molecular weight distribution of polymer macromolecules, they have different distribution coefficients in solution. Such a distribution is conveniently represented by dynamic light scattering analysis results as a distribution curve with a number of fractions that are characterized by specific diffusion coefficients. Eq. 19 was solved for each fraction according to DLS data. The respective concentration distributions of each fraction in a microchannel were then combined into the total concentration distribution of PDADMAC in the main channel.

Eq. 19 with the boundary conditions represented by Eq. 22 and Eq. 23 was solved in Matlab. The script with the respective Matlab code is demonstrate below.

Matlab Script for Numerical Simulation of Polymer Diffusion

```
%THE SCRIPT FOR CONVECTION-DIFFUSION EQUATION OF PDADMAC POLYMER IN A
MICROFLUIDIC CHANNEL.

%First set global variables to be used by all the functions in this
script:
global k p Fraction Size D D0 H L U Q1 Q2 y asp;

%INPUT THE VALUES, WHICH CHARACTERIZE THE MICROFLUIDIC SYSTEM:

% Input channel diameter D0, µm; channel height, H, µm; and channel
length, L, mm:
D0=300; H=105; L=16;
asp = D0/H;

%Input height over the microchannel bottom, µm:
y = 15;

%Set distance from the junction point to extract solution for the
channel beginning, mm:
L1 = 3;
L2 = L/L1;

%Input the flowrate of polyelectrolyte Q1 and buffer Q2, µl/min:
Q1=.33; Q2=.67;

%Calculate the flow velocity U, mm/s:
U=(Q1+Q2)/(D0*H)*10^6/60;

%Calculate diffusion coefficients of polyelectrolyte from the PDADMAC
DLS data:
Size =
[4.19;4.85;5.61;6.5;7.53;8.72;10.1;11.7;13.5;15.7;18.2;21;24.2;28.2;32
.7;37.8;42.8;50.7;58.8;68.1];
Fraction =
[0.12;6.34;19.5;25.7;20.9;13.2;7.26;3.68;1.78;0.843;0.393;0.18;0.0814;
0.0358;0.0153;0.00629;0.00248;0.000932;0.00033;0.00011].*0.01;
kb = 1.3806488*10^-23;
T = 298;
n = 8.9*10^-4;
Diam = Size;
D = kb*T/(6*pi*n*0.5*10^-9)*10^12./Diam;

%Define convection-diffusion equation and solve it for each diffusion
coefficient:
k = ones(1,length(Size))';
p = zeros(1,length(Size))';

b=1;
a=1;
m = 0;
mesh = 100;
x = linspace(0,a,mesh);
```

```
t = linspace(0,b,mesh);

sol = pdepe(m,@pdex4pde,@pdex4ic,@pdex4bc,x,t);

%Combine solutions for each diffusion coefficients into a single
concentration distribution and extract solutions for the main channel
beginning and end:
u = sol(round(mesh/L2),:,:);
[~,r,c] = size(u);
u = u(:,:);
u = reshape(u,[r,c])';

contrib = Fraction.*u;
tot = sum(contrib);

u1 = sol(end,:,:);
[~,r1,c1] = size(u1);
u1 = u1(:,:);
u1 = reshape(u1,[r1,c1])';

contrib1 = Fraction.*u1;
tot1 = sum(contrib1);

[p2,r2,c2] = size(sol);
contrib2 = zeros(p2,r2,c2);
for l = 1:size(Fraction)
contrib2(:,:,l) = sol(:,:,l).*Fraction(l);
end

tot2 = sum(contrib2,3);

%Plot the calculation results:
figure
f = plot(x,tot);
f.LineWidth = 1;
f.Color = 'k';

figure
f1 = plot(x,tot1);
f1.LineWidth = 1;
f1.Color = 'r';

figure
surf(tot2)
shading interp
hold on
sc = surfc(tot2,'FaceAlpha',0, 'EdgeAlpha',0);
sc(2).EdgeColor = 'w';
sc(2).LineWidth = 2;
hcb=colorbar;
hcb.Location = 'east';
set(hcb,'YTick',[])
colormap(parula)
view(90,-90)
```

```
axis equal
axis off

%Introduce PDE coefficients and the boundary conditions:
% --------------------------------------------------------------
function [c,f,s] = pdex4pde(x,~,~,DuDx)
global k D D0 L p U y asp;
s = 1.7 + 0.5*(1/asp)^0.25;
r = 2;
Umax = (1+1/s)*(1+1/r);
c = Umax*(1-(2.*x-1)^2).*(1-(2.*y/100-1)^2).*U*D0*D0/L.*k;
f = D.* DuDx;
s = p;
end
% --------------------------------------------------------------
function u0 = pdex4ic(x)
global Q1 Q2 k
in = x<=Q1/(Q1+Q2);
u0 = in.*k;
end
% --------------------------------------------------------------
function [pl,ql,pr,qr] = pdex4bc(~,ul,~,ur,~)
global k;
pl = ul(k);
ql = k;
pr = ur(k);
qr = k;
end
```

References

1. Sørensen, B. E., A revised Michel-Lévy interference colour chart based on first-principles calculations. European Journal of Mineralogy 2013, 25 (1), 5-10. Doi: 10.1127/0935-1221/2013/0025-2252.
2. Linge Johnsen, S. A.; Bollmann, J.; Lee, H. W.; Zhou, Y., Accurate representation of interference colours (Michel-Levy chart): from rendering to image colour correction. J Microsc 2018, 269 (3), 321-337. Doi: 10.1111/jmi.12641.
3. Chang, R., The Anisotropic Refractive Indices of Aligned MBBA Liquid Crystal Films. Molecular Crystals and Liquid Crystals 2007, 28 (1-2), 1-8. Doi: 10.1080/15421407408083148.
4. Berthier, J.; Silberzan, P., Microfluidics for Biotechnology, Second Edition. Artech House: London, 2009; p 512.
